# Cold Acclimation Improves the Desiccation Stress Resilience of Polar Strains of *Klebsormidium* (Streptophyta)

**DOI:** 10.3389/fmicb.2019.01730

**Published:** 2019-08-06

**Authors:** Martin Rippin, Nadine Borchhardt, Ulf Karsten, Burkhard Becker

**Affiliations:** ^1^Department of Biology, Botanical Institute, University of Cologne, Cologne, Germany; ^2^Department of Biology, University of Rostock, Rostock, Germany

**Keywords:** cold acclimation, desiccation stress, *Klebsormidium*, polar regions, transcriptomics

## Abstract

Biological soil crusts (BSCs) are complex communities of autotrophic, heterotrophic, and saprotrophic (micro)organisms. In the polar regions, these biocrust communities have essential ecological functions such as primary production, nitrogen fixation, and ecosystem engineering while coping with extreme environmental conditions (temperature, desiccation, and irradiation). The microalga *Klebsormidium* is commonly found in BSCs all across the globe. The ecophysiological resilience of various *Klebsormidium* species to desiccation and other stresses has been studied intensively. Here we present the results of transcriptomic analyses of two different *Klebsormidium* species, *K. dissectum* and *K. flaccidum*, isolated from Antarctic and Arctic BSCs. We performed desiccation stress experiments at two different temperatures mimicking fluctuations associated with global change. Cultures grown on agar plates were desiccated on membrane filters at 10% relative air humidity until the photosynthetic activity as reflected in the effective quantum yield of photosystem II [Y(II)] ceased. For both species, the response to dehydration was much faster at the higher temperature. At the transcriptome level both species responded more strongly to the desiccation stress at the higher temperature suggesting that adaptation to cold conditions enhanced the resilience of both algae to desiccation stress. Interestingly, the two different species responded differently to the applied desiccation stress with respect to the number as well as function of genes showing differential gene expression. The portion of differentially expressed genes shared between both taxa was surprisingly low indicating that both *Klebsormidium* species adapted independently to the harsh conditions of Antarctica and the Arctic, respectively. Overall, our results indicate that environmental acclimation has a great impact on gene expression and the response to desiccation stress in *Klebsormidium*.

## Introduction

Biological soil crusts (BSCs) are the dominant vegetation cover on the temporarily snow- and ice-free soil surfaces of the Arctic and Antarctica (Yoshitake et al., 2010; Williams et al., 2017). These communities are complex agglomerations formed by diverse autotrophic and heterotrophic organisms such as microalgae, lichens, bryophytes, fungi, bacteria, and micro fauna (Belnap, 2006; Darby and Neher, 2016). The streptophyte green microalga *Klebsormidium* (Klebsormidiophyceae) is commonly associated with BSCs in both the Arctic and Antarctica (Hayashi and Shinozaki, 2012; Pushkareva et al., 2016; Borchhardt et al., 2017a,b; Rippin et al., 2018). In general, *Klebsormidium* is one of the most widespread green algal genera and can be found in numerous habitats around the globe (Elster et al., 2008; Škaloud and Rindi, 2013). Members of this genus are poikilohydric and, hence, unable to actively regulate their cellular water content which in turn may lead to nearly complete dehydration upon desiccation (Holzinger et al., 2014; Karsten and Holzinger, 2014).

The polar regions are characterized by a pronounced seasonality, low water availability for most of the year, and generally low temperatures resulting in harsh environmental conditions forcing organisms to develop adaptive mechanisms. The desiccation tolerance of *Klebsormidium* had already been described 100 years ago by Piercy (1917) and its ecophysiology has been widely studied on many biogeographically diverse isolates (e.g., Holzinger et al., 2014; Herburger and Holzinger, 2015; Blaas and Holzinger, 2017; Donner et al., 2017). Elster et al. (2008) showed that Arctic and Antarctic *Klebsormidium* strains are resistant to desiccation-induced injuries. Holzinger et al. (2014) studied the underlying molecular mechanisms in an alpine isolate of *Klebsormidium crenulatum* and found that dehydration caused an inhibition of photosynthesis while photosynthetic gene transcripts were up-regulated to prepare for rehydration. Furthermore, pathways leading to the synthesis of sucrose and raffinose, both important osmolytes, were up-regulated (Holzinger et al., 2014). Increasing the amount of osmolytes in the cell leads to a more negative osmotic potential which helps to retain water within the cell and protects against membrane damage and protein aggregation (Bisson and Kirst, 1995).

In addition, *Klebsormidium* enhances the expression of heat-shock proteins (HSPs) and chaperones [e.g., late embryogenesis abundant (LEA) proteins] to prevent protein aggregation and misfolding (Holzinger et al., 2014). As dehydration also enhances the formation of harmful reactive oxygen species (ROS), organisms have to activate appropriate scavenging mechanisms such as the synthesis of antioxidants (Cruz de Carvalho, 2008). Indeed, the desiccation transcriptome of *K*. *crenulatum* suggests an accumulation of the antioxidant glutathione and carotenoids in response to water withdrawal (Holzinger et al., 2014).

Besides low water availability, polar strains of *Klebsormidium* also have to endure low temperatures and long periods of freezing. Manabu et al. (2008) analyzed cold-acclimated filaments of *K. flaccidum* and found that this microalga had accumulated soluble sugars, such as glucose and sucrose, free amino acids, such as γ-aminobutyric acid, as well as starch grains and oil droplets. Changes in carbohydrate homeostasis may also enable the alga to cope with elevated ROS levels induced by low temperatures (Pommerrenig et al., 2018). Moreover, this species modified its cellular structure, reduced the size of its vacuole, and enlarged both its chloroplasts and cytoplasm (Manabu et al., 2008). Molecular data on the cold-responsive pathways and coping mechanisms are still lacking for *Klebsormidium*. However, studies on other algae exposed to low temperatures are available. Valledor et al. (2013) investigated the lipid profiles in the chlorophyte *Chlamydomonas reinhardtii* upon cold stress and found an increase in polyunsaturated fatty acids that is likely occurring to maintain membrane fluidity. The streptophyte *Spirogyra varians* (Han et al., 2012) accumulated antioxidants at 4°C suggesting an accelerated ROS generation (Han et al., 2012).

*Klebsormidium* plays an important role in the formation of BSCs, owing to its filamentous morphology and exopolysaccharide production, and also contributes to the primary production of the crust (Barberousse et al., 2006; Büdel et al., 2016). In general, biological crusts are essential components of polar ecosystems as they fix nitrogen and carbon dioxide, protect the soil against erosion, and enhance its fertilization (Evans and Johansen, 1999). However, climate change poses a major threat to these communities as it may affect their composition and abundance substantially (Johnson et al., 2012; Zelikova et al., 2012). For example in Antarctica, permanently ice- and snow-covered ground sets natural species boundaries (Lee et al., 2017). With increasing temperatures these zones will expand and eventually amalgamate which could offset those limits (Lee et al., 2017). Besides warming, higher levels of radiation and altered precipitation patterns are predicted for the Arctic and Antarctica, in particular the Antarctic Peninsula, which may cause an invasion of foreign species (Chown et al., 2012; Pushkareva et al., 2016). Populations of natives, that are unable to compete with those alien species, are likely to become diminished or even die (Bellard et al., 2016).

Here we present a transcriptomic study on two species of *Klebsormidium*, isolated from Arctic Svalbard and Ardley Island, Antarctica. Both taxa were tested for their desiccation tolerance. We cultivated both species at 5 and 20°C prior to treatment to elucidate how temperature influences the organism’s ability to cope with dehydration stress. Afterward, the filaments were rewetted with water and allowed to recover. Transcriptomes were analyzed at two time points during recovery to assess the underlying molecular mechanisms.

## Materials and Methods

### Algal Isolates and Cultivation

Two culture isolates of *Klebsormidium* were used in this study: *K. dissectum* (EiE-15a; Borchhardt et al., 2017a) was isolated from a BSC collected at Svalbard, Norway, and *K. flaccidum* (A1-1a; Borchhardt et al., 2017b) was isolated from an Antarctic BSC sampled at Ardley Island, South Shetland Islands. *K. dissectum* belongs to the *Klebsormidium* clade E in the clade system, described by Mikhailyuk et al. (2015), while *K. flaccidum* is affiliated with clade B/C. Both strains are part of the Culture Collection at the University of Rostock.

Both microalgae were cultured on solid 1.5% Difco^TM^ Agar (Becton Dickinson GmbH, Heidelberg, Germany) enriched with Bold’s basal medium supplemented with vitamins and a tripled nitrate concentration (3N-BBM+V, Starr and Zeikus, 1993) at both 5 and 20°C, and 35 μmol photons m^–2^ s^–1^ (Daylight Lumilux Cool White Lamps L36W/840, OSRAM Licht AG, Munich, Germany) under a 16:8-h light–dark cycle.

### Growth in Response to Temperature

*In vivo* chlorophyll *a* fluorescence was used as a proxy for biomass accumulation according to Gustavs et al. (2009). Growth experiments were carried out at 12 different temperatures (6, 8, 10, 12, 13, 14, 17, 18, 20, 23, 26, and 28°C) at 35 μmol photons m^–2^ s^–1^ and a 16:8-h light–dark cycle using a self-designed algal incubator (Kunststoff-Technik GmbH, Rostock, Germany) as described in Woelfel et al. (2014). For acclimation purposes all measurements were always done with log-phase cultures that were pre-incubated at experimental conditions for 10 days. Chlorophyll *a* fluorescence (excitation 440 nm, emission 680 nm) was measured each day with a SpectraMax M2e multiplate reader (MPR; Molecular Devices, Biberach, Germany) using the software SoftMax Pro version 5.4 (Molecular Devices, LCC, San Jose, CA, United States). Increasing fluorescence was detected as relative fluorescence units (RFUs) and the fluorescence measured directly after the inoculation serving as a starting value. Before each measurement, a dark-incubation of 10 min was performed in order to open all reaction centers of photosystem (PS) II. Calculation of growth rate for each individual replicate was performed as described in Gustavs et al. (2009).

### Desiccation Treatment and Rehydration

Both species were cultivated in triplicates in liquid cultures for 10 days under a light regime of 35 μmol photons m^–2^ s^–1^ and a 16:8-h light–dark cycle (log-phase) at 5 or 20°C. Following cultivation, 15 ml of each culture was blotted onto Whatman^TM^ GF six glass fiber filters (GE Healthcare, Little Chalfont, United Kingdom). Three filters of each species and temperature were directly harvested (C = control). The remaining filters were placed into desiccation chambers, developed by Karsten et al. (2014), and dried over 100 g of activated silica gel (Carl Roth, Karlsruhe, Germany), which led to a constant, relative air humidity of about 10%. Desiccation experiments were carried out at 5 or 20°C. During treatment, the effective quantum yield of PS II [Y(II)] was monitored using the pulse amplitude modulated fluorometer PAM 2500 (Heinz Walz, Effeltrich, Germany). Once Y(II) reached zero, another triplicate of filters was sampled (D = desiccated). Immediately afterward, the silica gel was replaced by 100 ml of tap water and the dried filaments were wetted with 200 μl of medium. The relative air humidity in the chamber was now between 95 and 98%. Filter triplicates were harvested 2 h after the wetting (RI = rehydration I) and 24 h after the start of the experiment (RII = rehydration II).

### RNA Isolation and Sequencing

All harvested filters were instantly quenched in liquid nitrogen and homogenized in a chilled mortar. Subsequently, the powder was processed with the Spectrum Kit (Sigma–Aldrich, St. Louis, MO, United States) according to Rippin et al. (2016). The quality of the resulting RNA samples was assessed on a BioAnalyzer instrument (Agilent Technologies, Santa Clara, CA, United States) and subsequently used to prepare libraries. Eukaryotic mRNA was enriched by means of oligo-(dT) beads, and fragmented and reverse-transcribed into cDNA using random hexamers. Prior to sequencing on an Illumina HiSeq (2 × 75 bp) at the Cologne Center for Genomics, appropriate adapters were ligated. All 48 libraries were multiplexed to eliminate batch effects during sequencing. All raw reads were submitted to the SRA database under the BioProject ID PRJNA500592.

### Bioinformatic Workflow

All raw reads were quality-trimmed and filtered using Trimmomatic 0.35 (Bolger et al., 2014) and PrinSeq Lite 0.20.4 (Schmieder and Edwards, 2011). rRNA sequence reads were separated by SortMeRNA 2.1 (Kopylova et al., 2012) using SILVA SSU NR Ref 119 and LSU Ref 119. Before assembling the reads, COPE 1.2.5 (Liu et al., 2012) was utilized to stitch overlapping reads together. The assembly of the *K. dissectum* and *K*. *flaccidum* transcriptomes was done with Trinity 2.0.6 (Grabherr et al., 2011) and the quality was assessed with scripts from the Trinity package and BUSCO 3.0.2 combined with the embryophyta database (Simão et al., 2015). The assemblies were annotated using the Trinotate pipeline 3.0.0^1^, including TransDecoder 2.1^2^, NCBI BLAST+ 2.3.0 (Altschul et al., 1990), HMMER 3.1 b (Finn et al., 2011), SignalP 4.1 (Petersen et al., 2011), TMHMM 2.0 c (Krogh et al., 2001), RNAmmer 1.2 (Lagesen et al., 2007), and the databases Swiss-Prot (Bairoch and Apweiler, 1999), PFAM 3.1b2 (Sonnhammer et al., 1997), RefSeq Plant 149, the transcriptomes of *K. crenulatum* (Holzinger et al., 2014) and *K. subtile* from the 1KP data repository (Matasci et al., 2014) as well as the genome of *K. nitens* (Hori et al., 2014). The expect value cutoff was set to *E* −10. The gene map plot was created using the *R* package *circlize* (Gu et al., 2014).

The raw reads, obtained from the different conditions tested, were mapped onto the assembled contigs of both organisms using Bowtie 1.1.2 (Langmead et al., 2009) and abundance was estimated with RSEM 1.2.30 (Li and Dewey, 2014). Subsequently, the *R* package edgeR (Robinson et al., 2010) was employed to identify differentially expressed genes. Genes possessing an FDR (Hochberg, 1995) of >0.001 and a fold change of <4 were excluded from further analysis. Overlapping gene sets between the same comparisons at different temperatures were tested for significance using a hypergeometric distribution in *R*. Two types of gene set enrichment analyses were carried out in *R*: GO term enrichment with GoSeq 1.26.0 (Young et al., 2010) and Kyoto Encyclopedia of Genes and Genomes (KEGG) pathway enrichment with clusterProfiler 3.2.11 (Yu et al., 2012), setting the FDR in both cases to 0.05. The online tool REVIGO (Supek et al., 2011) was used to create the basis data for the GO network plots.

## Results

### Ecophysiological Analysis

**FIGURE 1:**
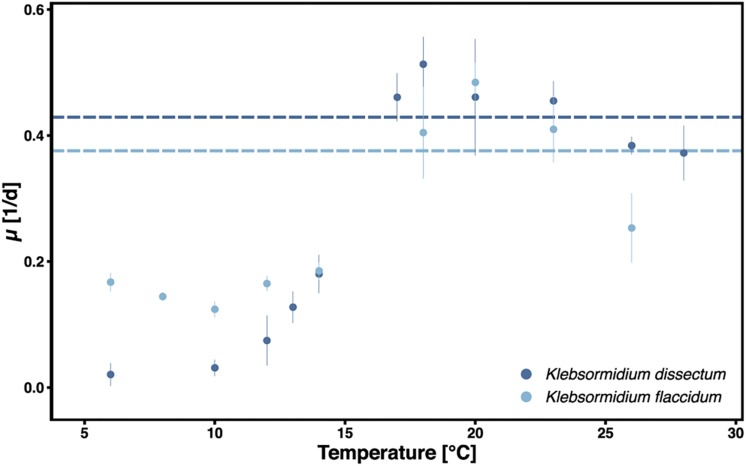
Growth of *K. dissectum* and *K. flaccidum* at different temperatures. The growth rate μ was determined for exponential cultures. Bars indicate standard deviation. Horizontal lines indicate the mean maximal growth rate between 12 and 28°C.

*Klebsormidium dissectum* and *K. flaccidum* exhibited biomass accumulation between 6 and 28°C (Figure 1). The optimal growth temperature was 18 and 20°C for *K. dissectum* and *K. flaccidum*, respectively, which classifies both taxa as psychrotolerant. At the optimal temperatures both strains divided every 2.5 days. However, *K. dissectum* growed very slowly at low temperatures, with a generation time of approximately 20 days at 6 and 10°C. In contrast, *K. flaccidum* cells divided on average every 5–10 days between 6 and 15°C.

**FIGURE 2:**
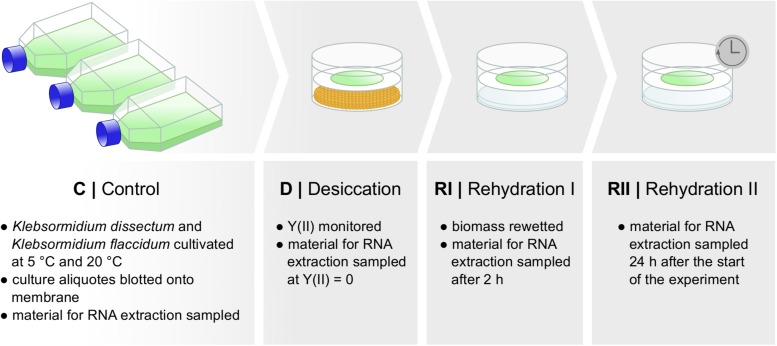
Experimental set up. Two different species of *Klebsormidium* were desiccated at two different temperatures [5 and 20°C until Y(II) reached zero] for the time indicated in Figure 3 and allowed to recover for 2 h and approximately 24 h. Samples were taken at several time points for control, desiccation treatment, and during the recovery phase. RNA was subsequently extracted.

**FIGURE 3:**
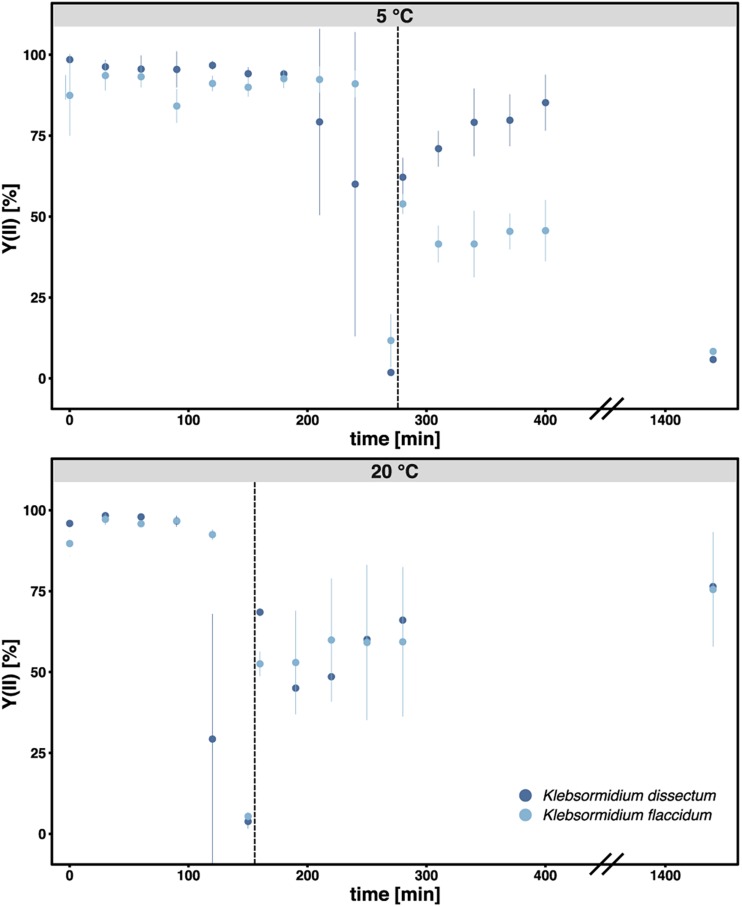
The photosynthetic quantum yield of PS II [Y(II)] was measured over time during the course of desiccation for all samples. Each triplicate was measured three times at different positions of the filter. The bar indicates standard deviation while the dots indicate the mean value for each treatment. Dark blue: *K. dissectum*; light blue: *K. flaccidum*. The vertical lines indicate the start of the recovery phase.

Figure 2 shows the experimental setup of the desiccation experiment. As in earlier studies (Holzinger et al., 2014; Rippin et al., 2017), we monitored the desiccation process indirectly by measuring the photosynthetic quantum yield of PS II [Y(II)] with a pulse amplitude modulated fluorometer (PAM). For both taxa, *K. flaccidum* and *K. dissectum*, the desiccation time was similar at the same temperature (Figure 3). At 5°C, it took about 270 min to record a minimum Y(II) = 0, while at 20°C the time required to reach a Y(II) = 0 decreased to approximately 150 min. A partial recovery of Y(II) (50–80%) was observed 24 h after the start of the experiment for both *Klebsormidium* species. While the recovery of *K. flaccidum* was independent from temperature (approximately 50%), *K. dissectum* showed a better recovery at 5°C (approximately 75%) compared to 20°C (approximately 50%).

### Sequencing Outcome and Assembly

About 5.7 ± 0.57 Gbp, which corresponds to approximately 38 ± 4 Mio read pairs (Supplementary Table S1), per library were successfully sequenced. After quality trimming and filtering, 14 ± 6.4 Mio read pairs per library remained and were assembled to 91,678 and 99,957 contigs for *K. dissectum* and *K. flaccidum*, respectively. The N50 value for *K. dissectum* was 2,805 bp and for *K. flaccidum* 3,021 bp.

**FIGURE 4:**
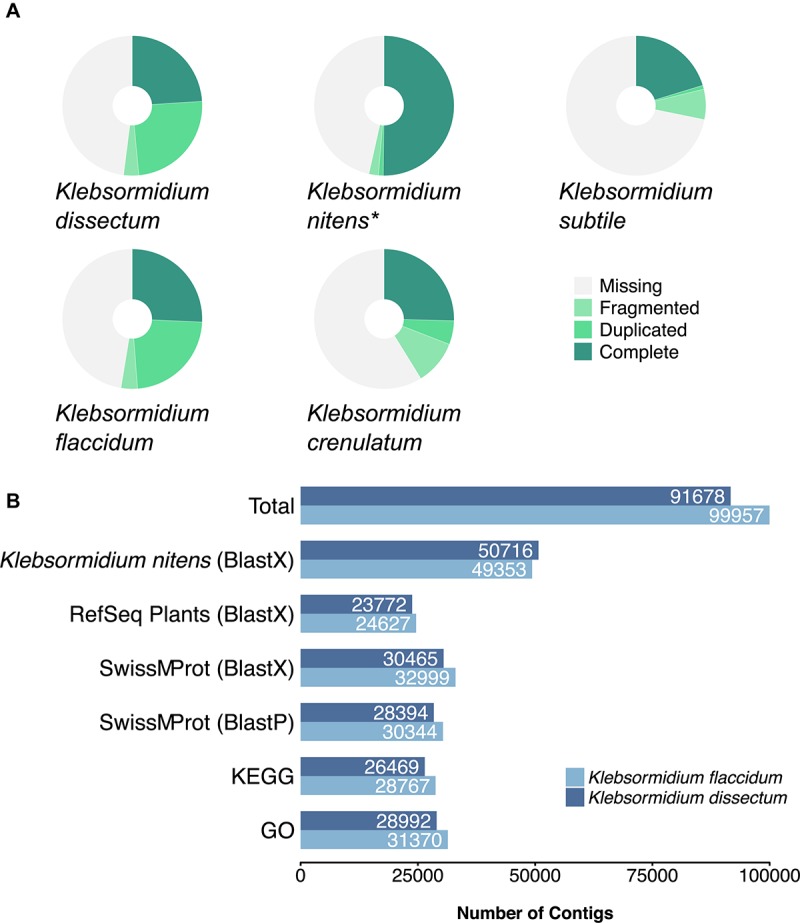
**(A)** BUSCO analysis of the assembled transcriptomes from *K. dissectum* and *K. flaccidum*. The pie chart displays the results of the BUSCO analysis with the categories complete, duplicated, fragmented, and missing. For comparison the transcriptomes of *K. crenulatum* and *K. subtile*, as well as the genome (indicated with an asterisk) of *K. nitens* were included in the BUSCO analysis. **(B)** Summary of the annotation results for *K. dissectum* and *K. flaccidum*. The assembled contigs were blasted against the genome of *K. nitens* and the indicated databases using the indicated BLAST program and an *e*-value cut-off of *E* –10.

A BUSCO analysis was performed for both assemblies as well as for the genome of *K. nitens*, the transcriptome of *K. crenulatum* and the transcriptome of *K. subtile* for comparison (Figure 4A). In the case of *K. dissectum*, 48.5% of the universal single-copy orthologs was found to be complete (either single or duplicated), 3.5% was fragmented, and 48% was missing. The situation was similar for *K. flaccidum* with 48.8% of the BUSCO orthologs to be complete, 3.8% fragmented, and 47.4% missing. Running the same analysis on the genome of *K. nitens* revealed that both assembled transcriptomes were in the same range as the genome. However, the large number of duplicated orthologs in the transcriptomes suggest that the transcriptomes probably still contain artificial isoforms, produced by the Trinity assembler that the BUSCO analysis classifies as duplications. The BUSCO ortholog coverage for the transcriptomes from *K. crenulatum* and *K. subtile* was lower when compared to *K. dissectum* and *K. flaccidum*.

**FIGURE 5:**
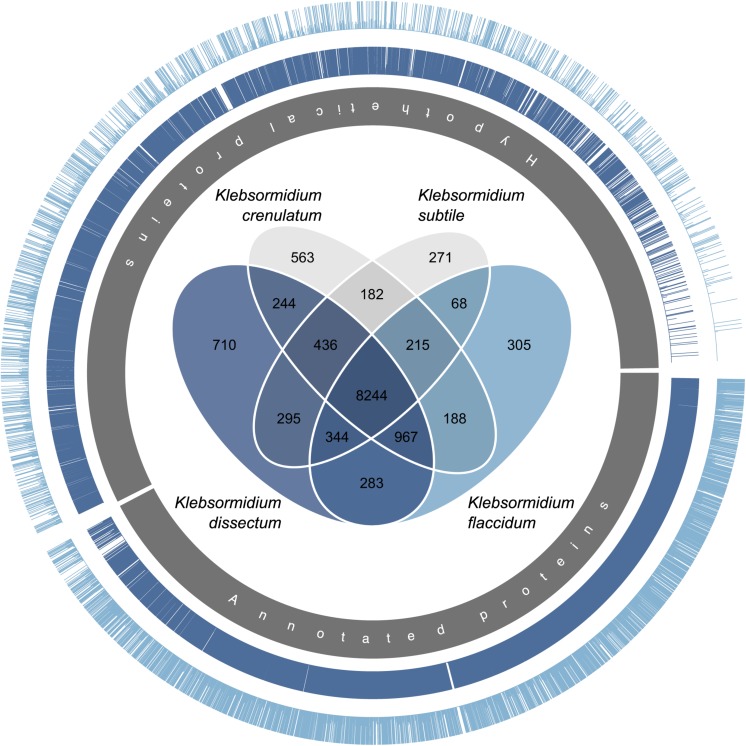
Gene map comparing the genome of *K. nitens* with the transcriptomes from *K. dissectum* and *K. flaccidum*. The inner gray circle indicates the annotated and hypothetical proteins of *K. nitens*. Each bar in the two outer circles represents a protein of *K. nitens* being a BLAST top hit when the two transcriptomes were blasted against the *K. nitens* genome. The bar length indicates the conservation of the protein. The proteins encoded by the *K. dissectum* transcriptome are generally much more similar to *K. nitens* proteins than proteins encoded the *K. flaccidum* transcriptome. The Venn diagram in the center illustrates the similarity of the available *Klebsormidium* transcriptomes when the transcriptomes were blasted against the *K. nitens* genome using the BLASTN algorithm and an *e*-value cut-off of *E* –10.

Both of the assembled transcriptomes were annotated against Swiss-Prot and RefSeq Plant databases with annotation rates from 24.6 to 33.2% (Figure 4B). The annotation against the genome of *K. nitens* resulted in 55 and 49.4% identified transcripts. The gene map in Figure 5 shows the nucleotide sequence similarities of both species to each protein of *K. nitens*. The expect values and coverage for *K. dissectum* were generally higher than for *K. flaccidum*. The Venn diagram in the center shows the BlastN results of the transcriptomes of *K. dissectum*, *K. flaccidum*, *K. crenulatum*, and *K. subtile* against *K. nitens*. All transcriptomes shared a total of 2,665 genes with the *K. nitens* genome at the mRNA level with an expect value threshold of at least *E* −10. The transcriptomes of *K. dissectum* and *K. crenulatum* shared the largest and smallest fraction with *K. nitens*, respectively.

Moreover, the assembled transcriptomes were annotated for KEGG IDs 17.4 and 12.1% of the *K. dissectum* contigs were successfully assigned KO (KEGG orthology) terms and *Arabidopsis* identifiers, respectively. For *K. flaccidum*, 17.6% of the assembled transcripts could be assigned KO terms and 13.3% *Arabidopsis* annotations. The KO terms from both assemblies were mapped onto the KEGG metabolic pathway map (ko01100). The assembled transcriptomes of *K. dissectum* and *K. flaccidum* showed a good coverage of the most important pathways (e.g., carbohydrate metabolism, amino acid metabolism, fatty acid metabolism, nucleotide metabolism, respiration) as illustrated in Supplementary Figures S1, S2. For functional characterization, GO (gene ontology) terms were assigned which yielded annotation rates of 31.6 and 31.4% for *K. dissectum* and *K. flaccidum*, respectively (Figure 4B).

### Differential Gene Expression

**FIGURE 6:**
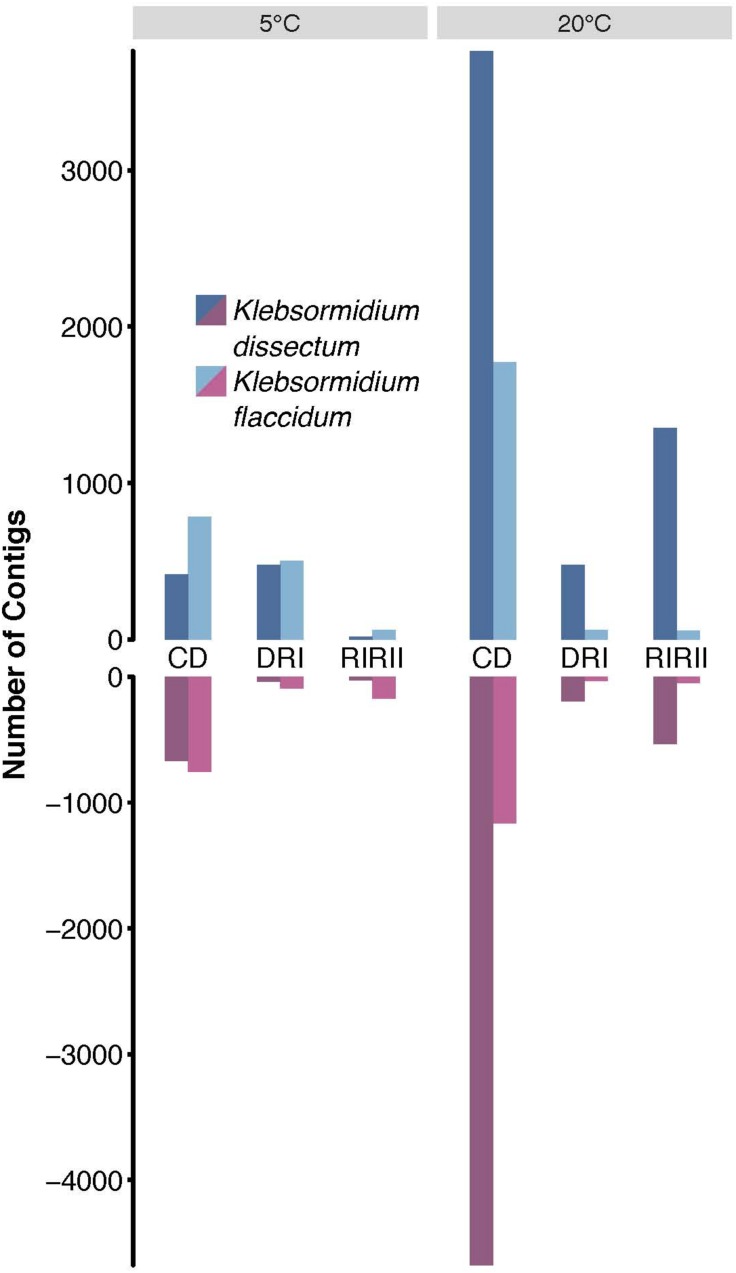
Differential gene expression analysis. The total of upregulated (dark and light blue) and downregulated (dark and light red) contigs of *K. dissectum* and *K. flaccidum* upon desiccation and during recovery at 5 and 20°C. Only contigs with a FDR of less or equal to ≤0.001 and a fold change of at least >4 were considered.

To assess which genes were differentially expressed, the raw reads of all libraries were mapped onto the assembled transcripts with a mapping efficiency of 98.99 ± 0.25% (Supplementary Table S1). Figure 6 shows that the overall strongest response for both species was triggered by the desiccation treatment at 20°C while the weakest response was observed for the second rehydration period at 5°C. Furthermore, *K. dissectum* responded much stronger at 20°C to all treatments compared to *K. flaccidum*.

The largest overlap of differentially expressed transcripts at 5 and 20°C was observed for the comparison between controls to desiccated samples (Table 1). For *K. dissectum*, 204 and 329 genes were up- and down-regulated at both temperatures, respectively. *K. flaccidum*, on the other hand, increased the transcript pool of 311 genes and repressed 164 genes at both temperatures. Comparing desiccation and the first time point of rehydration, *K. dissectum* shared 66 up-regulated genes between the two temperatures while *K. flaccidum* exhibited an overlap of 16 up-regulated transcripts. Otherwise, no significant overlap was found.

**TABLE 1:** Number of regulated genes shared between different treatments.

**Organism**	**Pair1**	**Pair2**	**Direction**	**Number of shared transcripts**	**HyperP**
*K. dissectum*	20_C_D	5_C_D	Up	204	4.17*E*−166
*K. dissectum*	20_C_D	5_C_D	Down	329	1.41E−237
*K. flaccidum*	20_C_D	5_C_D	Up	311	0
*K. flaccidum*	20_C_D	5_C_D	Down	164	1.27*E*−155
*K. dissectum*	20_D_HI	5_D_HI	Up	66	2.06*E*−72
*K. dissectum*	20_D_HI	5_D_HI	Down	1	0.08009545
*K. flaccidum*	20_D_HI	5_D_HI	Up	16	2.12*E*−23
*K. flaccidum*	20_D_HI	5_D_HI	Down	1	0.02783477
*K. dissectum*	20_HI_HII	5_HI_HII	Up	0	1
*K. dissectum*	20_HI_HII	5_HI_HII	Down	2	0.01075614
*K. flaccidum*	20_HI_HII	5_HI_HII	Up	1	0.03361651
*K. flaccidum*	20_HI_HII	5_HI_HII	Down	0	1

### Gene Set Enrichment Analysis

For KEGG enrichment analysis, *Arabidopsis* IDs were used and the results are displayed in Table 2. *K. dissectum* showed almost exclusively all enrichments at 20°C while *K. flaccidum* solely exhibited enriched pathways at 5°C. Upon desiccation, the down-regulated transcripts of *K. dissectum* showed an enrichment for the aminoacyl-tRNA biosynthesis, DNA replication, and photosynthesis while the ether lipid metabolism, the fatty acid degradation, the glycerophospholipid metabolism, and the butanoate metabolism were enriched in the up-regulated fraction of the transcriptome. At 5°C, *K. flaccidum* exhibited enrichment of the glycine, serine, and threonine metabolism in the down-regulated contigs during dehydration.

**TABLE 2:** A KEGG pathway enrichment analysis based on KO annotations was performed for the up- and downregulated transcripts in all analyzed groups using the ath_pathway map at KEGG.

**Pathway ID**	**Pathway description**	***p*-value**	***p*-adjust**	***q*-value**	**Temperature (°C)**	**Comparison**
***K. dissectum***						
ath00970	Aminoacyl-tRNA biosynthesis	6.41*E*−05	0.00331476	0.00310626	20	C-D Down
ath03030	DNA replication	8.08*E*−05	0.00331476	0.00310626	20	C-D Down
ath00195	Photosynthesis	0.00118163	0.03229795	0.03026637	20	C-D Down
ath00565	Ether lipid metabolism	3.10*E*−05	0.00235433	0.00202172	20	C-D Up
ath00071	Fatty acid degradation	0.00015319	0.00582105	0.00499868	20	C-D Up
ath00564	Glycerophospholipid metabolism	0.0017691	0.0448172	0.03848569	20	C-D Up
ath00650	Butanoate metabolism	0.00245153	0.04657909	0.03999866	20	C-D Up
ath04626	Plant–pathogen interaction	0.00170058	0.03231101	0.03222151	0	D-HI Down
ath03008	Ribosome biogenesis in eukaryotes	0.00033498	0.01507422	0.01410453	20	D-HI Up
ath00970	Aminoacyl-tRNA biosynthesis	5.98*E*−05	0.00370849	0.00358886	20	HI-HII Up
ath04626	Plant–pathogen interaction	0.00157064	0.00314128	0.0016533	5	HI-HII Down
***K. flaccidum***						
ath00260	Glycine, serine, and threonine metabolism	0.00115255	0.03227144	0.03154351	5	C-D Down
ath00592	alpha-Linolenic acid metabolism	0.00752394	0.01504788	0.00791994	5	HI-HII Down
ath03013	RNA transport	0.04035568	0.04035568	NA	5	HI-HII Up

**FIGURE 7:**
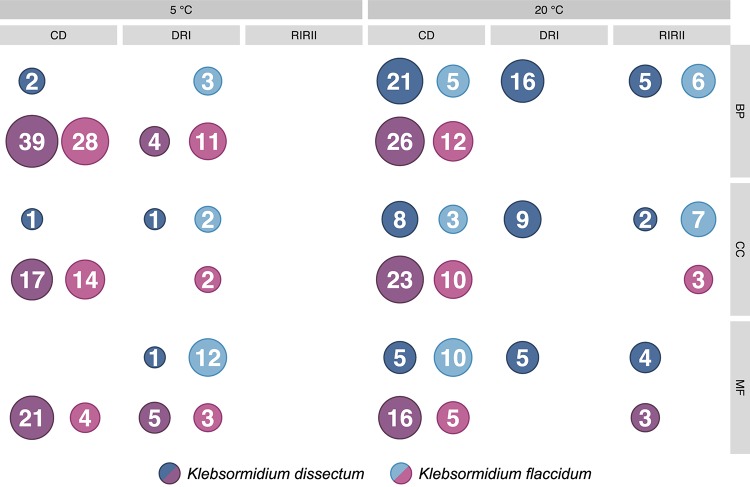
Summary of the GO enrichment analysis. The number of enriched categories, classified according to the three root categories, in the up- and down-regulated part of the transcriptomes are indicated. The detailed GO enrichment analysis is presented in Supplementary Table S2.

**FIGURE 8:**
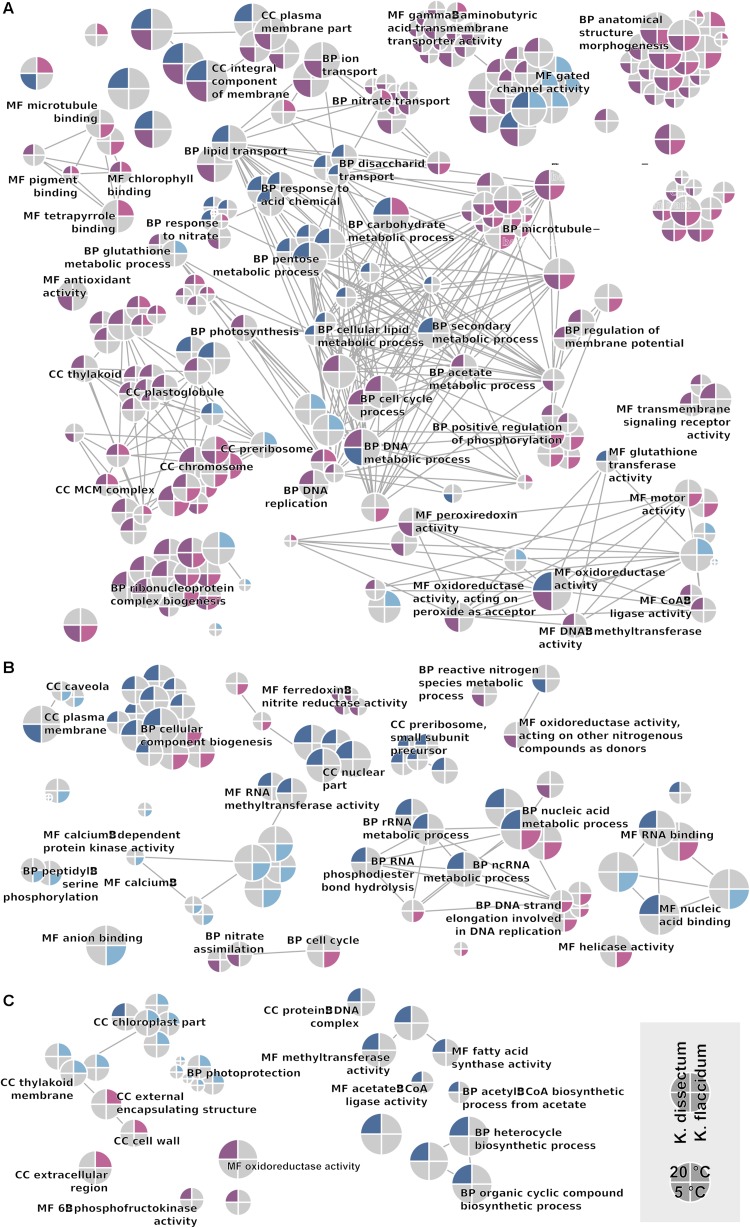
GO network displaying all enriched categories in both *Klebsormidium* species. Terms regulated at 5°C are indicated as the lower half of the circle, while terms regulated at 20°C are depicted in the upper half. Upregulation and downregulation are indicated with blue and red colors. *K. dissectum* is on the right while *K. flaccidum* is on the left side. The root categories are “biological process” (BP), “molecular function” (MF), and “cellular component” (CC). Edges depict shared higher categories. **(A)** Comparison of control cells with desiccated cells. **(B)** Comparison of desiccated cells with cell rehydrated for 2 h. **(C)** Comparison of cells with short (2 h) and long rehydration (24 h after start of the experiment) periods.

The GO term enrichment analysis (Supplementary Table S2) revealed a complex regulation during treatment in both *K. dissectum* and *K. flaccidum*. Figure 7 displays the number of enriched GO terms in the root categories (BP, biological process; CC, cellular component; MF, molecular function) within the up- and down-regulated fraction of the transcriptomes in response to desiccation and rehydration. Dehydration treatment caused most enrichment in all three categories while the different time points after rehydration only showed a smaller number of enriched terms or none at all. In Figure 8, the relationship between the enriched GO terms is depicted as a network. Both species exhibited an enrichment of photosynthetic and related terms in the repressed transcripts at 20°C when desiccated (Figure 8A). *K. dissectum* also showed a high number of terms belonging to membrane modification and the carbohydrate metabolism in the up-regulated fraction of the transcriptome at 20°C upon dehydration. The desiccation treatment at 5°C caused an enrichment of cytoskeleton associated terms in the repressed transcript pool of both species. Rehydration resulted in an enrichment of transcription and translation-related GO terms in *K. dissectum* at 20°C (Figure 8B). At 5°C, *K. flaccidum* exhibited enriched terms associated with signaling and replication in the up- and down-regulated differentially expressed genes, respectively. Figure 8C indicates accumulation of photosynthetic terms at 20°C in *K. flaccidum* during the second recovery period.

### Functional Context of Individual Genes

*Klebsormidium dissectum* showed a strong repression of photosynthetic transcripts at 20°C upon desiccation (Supplementary Table S3). Different parts of PS I and II, subunits of the light harvesting complex, and the magnesium chelatase subunit were down-regulated and partly up-regulated during the second recovery phase. In contrast at 5°C, *K. dissectum* exhibited almost no response in photosynthetic gene expression. The PS II oxygen-evolving enhancer protein 2 is 2.4-fold down-regulated upon water withdrawal. Moreover, the expression of early light-induced proteins (ELIPs) was elevated both during desiccation treatments as well as recovery at 20°C. At 5°C the same genes were down-regulated upon dehydration. *K. flaccidum* induced the transcription of ELIPs during the second recovery phase at 20°C and the desiccation treatment at 5°C. ELIP1a.2 was up-regulated fivefold at 20°C when the stress treatment was applied. In contrast to *K. dissectum*, the expression of photosynthetic genes appeared to be almost unaffected in *K. flaccidum*.

Both *Klebsormidium* species indicated a strong decline in cell cycle-associated transcripts when desiccated at 20°C (Supplementary Table S3). Affected genes included condensin, the spindle assembly checkpoint (SAC) protein, the leishmanolysin-like peptidase, the replication machinery in case of *K. dissectum*, the centromere-associated protein HEC1, the TPR domain containing protein, the pre-initiation complex, and the DNA helicase for *K. flaccidum* as well as several mini-chromosome maintenance proteins in both cases. At 5°C, the effect for both organisms was minor during desiccation treatment. In the first recovery phase, *K. flaccidum* represses the pre-initiation complex and some of the mini-chromosome maintenance proteins.

Transcription- and translation-related transcripts in *K. dissectum* showed a decrease at 20°C upon water stress while rewetting had an increasing effect on these processes (Supplementary Table S3). The U3 small nucleolar RNA-associated protein 10, a transcription elongation and a splicing factor, a RNA recognition motif containing protein, the ribosomal biogenesis regulatory protein, as well as multiple tRNA synthetases responded to the treatment and recovery with changes in their expression patterns. In contrast, *K. flaccidum* experienced an enhanced expression of an S1 RNA-binding domain containing protein, the U3 small nucleolar RNA-associated protein 24, a WD domain containing protein, and a N-terminal acetyltransferase at 20°C when exposed to dehydration.

At 20°C, a high number of genes belonging to the carbohydrate metabolism of *K. dissectum* were differentially expressed upon desiccation (Supplementary Table S3). This trend was not observed at 5°C neither for *K. flaccidum* at any temperature. Both α- and β-amylase, 4-α-glucanotransferase, hexokinase, phosphoglucomutase, sucrose phosphorylase, sucrose synthase, sucrose-phosphate synthase, and sucrose-phosphatase were up-regulated.

Changes in gene expression were also observed in the glycerophospholipid and glycerolipid pathway as well as the fatty acid metabolism for *K. dissectum* (Supplementary Table S3). This alga increased the transcript pool of phospholipase D, phosphoethanolamine *N*-methyltransferase, digalactosyldiacylglycerol synthase, diacylglycerol *O*-acyltransferase, and membrane-bound *O*-acyl transferase family protein at 20°C upon dehydration. The former two genes were also up-regulated at 5°C during desiccation treatment. Moreover, the expression of acyl-CoA oxidase and long-chain acyl-CoA synthase was enhanced at 20°C in response to dehydration while the acetyl-CoA carboxylase 1 was repressed. During the second recovery phase an approximately ninefold increase of acyl-CoA dehydrogenase and acetyl-CoA carboxylase 1 transcripts was detected.

Treatment affected cytoskeleton-related genes in both *K. dissectum* and *K. flaccidum* (Supplementary Table S3). Desiccation caused decrease in expression of actin-related protein Arp2/3 complex subunit C3 (p21), a calcium-binding actin-bundling protein, tubulin, γ-, δ-, and ε-tubulin in *K. dissectum* at 20°C. Calcium-binding actin-bundling protein, tubulin, and γ-tubulin were up-regulated during the second recovery phase. At 5°C, the transcript pool of ε-tubulin and β-tubulin folding cofactor C was reduced during water stress. Desiccation at 20°C also caused katanin p60 ATPase-containing subunit to become up-regulated. *K. flaccidum* showed a down-regulation of actin and related proteins, kinesin-like protein and dynamin family protein at 5°C during water withdrawal while the katanin p60 ATPase-containing subunit was up-regulated.

Several stress-related genes were regulated during the desiccation treatment and recovery (Supplementary Table S3). *K. dissectum* enhanced the expression of the following proteins: HSP70, chaperone DnaJ-domain superfamily protein, ATP-dependent chaperone ClpB, DNA repair protein, catalase, NADPH-dependent thioredoxin reductase, thioredoxin superfamily protein, dehydroascorbate reductase, glutathione-*S*-transferase (GST), glutathione transferase, early-responsive to dehydration stress (ERD) family protein, ERD4, LEA proteins, ζ-carotene desaturase, phytoene dehydrogenase, and carotenoid isomerase at 20°C upon desiccation. During the recovery and at 5°C, some of these genes showed complex regulation patterns. In *K. flaccidum*, HSP70, chaperone DnaJ-domain superfamily protein, ATP-dependent chaperone, catalase, peroxiredoxin, dehydroascorbate reductase, GST family protein, cupin superfamily protein, and LEA proteins also have complex expression patterns at both temperatures and during the different treatments.

## Discussion

### Genetic Similarity of the *Klebsormidium* Species

The two *Klebsormidium* species examined in this study were not closely related. The phylogeny/systematics of the genus *Klebsormidium* has been investigated in recent years and all known taxa can be grouped into 14 different clades (Škaloud and Rindi, 2013; Mikhailyuk et al., 2015). *K. dissectum* belongs to clade E, the largest clade of morphologically similar strains. This clade also includes the previously studied *K. nitens*, for which the genome is available (Hori et al., 2014), while the earlier studied *K. crenulatum* (Holzinger et al., 2014) belongs to clade F. The other investigated strain *K. flaccidum* belongs to clade B/C. Both transcriptomes showed considerable differences to each other when compared with the published genome. The transcriptome sequences obtained for *K. dissectum* were more similar to the published genome in terms of genome coverage and sequence similarity (Figure 5) than the sequences obtained for *K. flaccidum*. Other *Klebsormidium* transcriptomes available were from one other species within clade F (*K. crenulatum*, Holzinger et al., 2014) and within clade E (*K. subtile*, Matasci et al., 2014). Therefore, the two new transcriptomes reveal for the first time the intra-genus genomic variability within *Klebsormidium*.

In the following sections we will discuss various cellular processes and their responses to the applied desiccation stress and recovery phases. However, before looking at the different processes it might be import to point out that changes in the transcriptome do not cover the complete response of a cell to a changing environment. Pathways might be switched on or off completely posttranslationally and changes in the proteome might be due to changes in the mRNA concentration, translation efficiencies, and/or protein degradation rates (Payne, 2015). Thus, for a complete picture, we would additionally need the proteomes, metabolomes, etc. under the different conditions.

### Photosynthesis

Water is crucial for photosynthetic activity as it is necessary to maintain the structural integrity as well as the functionality of algal cells. Furthermore, water molecules play an essential role as electron donors in the electron transport chain of the photosynthesis. In the case of desiccation, photosynthetic organisms may exhibit one of three different coping strategies: escape, avoidance, or tolerance (Holzinger and Karsten, 2013; Fernández-Marín et al., 2016). *Klebsormidium* is a desiccation-tolerant organism which stalls photosynthetic activity during water stress and resumes the process as soon as water becomes available again (Elster et al., 2008; Karsten et al., 2013; Fernández-Marín et al., 2016). Our results from monitoring the effective quantum yield of PS II Y(II) for *K. dissectum* and *K. flaccidum* were in agreement with these studies as Y(II) dropped to zero during desiccation and rose again after water was supplied to the organisms. Moreover, we observed a temperature effect as it took longer to reach a Y(II) of zero at 5°C than at 20°C. The delayed photosynthetic inhibition at 5°C might have occurred due to the reduced water loss rates at lower temperatures (Yoder et al., 2010). However, the delay might also be explained by an increased dehydration tolerance which is caused by cold acclimation as both *Klebsormidium* species were cultivated at 5°C prior to the experiment. An increased desiccation tolerance resulting from an acclimation to lower temperatures has been described previously for higher plants (Cloutier and Siminovitch, 1982; Siminovitch and Cloutier, 1982; Koster and Bryant, 2006).

In response to desiccation at 20°C, *K. dissectum* repressed the expression of photosynthetic transcripts strongly while *K. flaccidum* did not respond as pronounced. In contrast, an alpine strain of *K. crenulatum* exhibited an up-regulation of photosynthetic genes upon desiccation at 20°C (Holzinger et al., 2014). Holzinger et al. (2014) argued that the increase in transcripts related to photosynthesis is a preparation for the recovery process after rehydration. However, the latter response is rather unusual. Carniel et al. (2016) compared a plethora of studies and found that most desiccation tolerant photosynthetic organisms, for example, the moss *Syntrichia ruralis* and the resurrection plant *Craterostigma plantagineum*, respond to water withdrawal by repression of genes involved in photosynthesis. The same holds true for the resurrection plant *Myrothamnus flabellifolia* which exhibits a down-regulation of transcripts encoding PSI and II probably to decrease the excitation energy and the associated ROS formation (Ma et al., 2015). This might also be the case for *K. dissectum* at 20°C. During the second phase of rehydration, *K. dissectum* reactivated the expression of those transcripts. The cyanobacterium *Anabaena* showed a similar recovery upon rewetting (Higo et al., 2007). Rather surprising is the observation that desiccation induced only minor changes in expression patterns regarding photosynthetic genes in *K. dissectum* at 5°C and in *K. flaccidum* at both temperatures. Hence, we assume that both *Klebsormidium* species cope with the water stress on different molecular levels, e.g., post-translational, which is invisible to expression studies.

Early light-induced proteins belong to the chlorophyll *a/b*-binding superfamily and act as photoprotectants upon abiotic stress, such as high light and ultraviolet (UV) radiation, but also desiccation (Zeng et al., 2002; Hutin et al., 2003; Hayami et al., 2015; Ma et al., 2015). These proteins are incorporated in the thylakoid membrane which they protect against photooxidative damage by binding free chlorophyll molecules and acting as sinks for excitation energy (Zeng et al., 2002; Hutin et al., 2003; Heddad et al., 2012; Hayami et al., 2015; Ma et al., 2015). *K. dissectum* at 20°C and *K. flaccidum* at 5°C exhibited an up-regulation of some ELIPs when dehydrated, most likely to protect the thylakoid membranes. Similar responses are known from other algae, such as the chlorophytes *C. reinhardtii* and *Dunaliella bardawil*, when exposed to high light stress (Lers et al., 1991; Teramoto et al., 2004). Other studies found that low temperatures induce ELIP expression too, for example, in the chlorophyte *Dunaliella salina* (Krol et al., 1997) and the streptophyte *S. varians* (Han and Kim, 2013). On the other hand, *K. dissectum* decreased the transcript pool of some ELIPs at 5°C upon desiccation treatment and *K. flaccidum* enhanced the expression of some ELIPs during the second phase of recovery at 20°C. The streptophytes *Zygnema circumcarinatum* (Rippin et al., 2017) and *K. crenulatum* (Holzinger et al., 2014) also showed a complex regulation of ELIPs when exposed to water stress which is typical for a multigene family (Zeng et al., 2002; Hutin et al., 2003).

### Cell Cycle

Several studies have described the inhibiting effect of water stress on cell division and cell cycle processes (e.g., Mansour and Hallet, 1981; Bagniewska-Zadworna, 2008; Kakumanu et al., 2012). Desiccation causes the moss *Polytrichum formosum* to arrest the mitotic phase upon completion and, consequently, all cells enter the interphase (Mansour and Hallet, 1981). An arrest of the cell cycle is also visible in the desiccation transcriptome of *K. crenulatum*. This alga repressed both DNA replication and the cell cycle upon water withdrawal (Holzinger et al., 2014). The same holds true for several transcripts of *K. dissectum* and *K. flaccidum* when these algae were desiccated at 20°C. Condensin, a chromosome condensation complex which is required to maintain the structural integrity of the chromosomes (Smith et al., 2014), is down-regulated in *K. dissectum*. Moreover, the transcript pool of the SAC protein is decreased. SAC proteins assure equal segregation of chromosomes during cell division (Komaki and Schnittger, 2017). Dehydration also caused a repression of transcripts that are part of the replisome and several DNA replication licensing factors (RLFs) in both *K. dissectum* and *K. flaccidum* at 20°C. These RLFs are essential to the cell as they limit the duplication of DNA to exactly once per cell cycle (Julian Blow and Chong, 1996). In addition, *K. flaccidum* exhibited a down-regulation of RLF transcripts at 5°C during the first recovery phase.

### Transcription and Translation

Severe desiccation can slow down and inhibit transcription and translation processes until water becomes available again (Farrant et al., 2007). At 20°C, *K. dissectum* showed signs of decelerated transcription and translation as a splicing factor and a number of aminoacyl-tRNA synthetases were down-regulated during dehydration treatment. Splicing is essential to convert pre-mRNA into mature mRNA (Chen and Cheng, 2012) while aminoacyl-tRNA synthetases are necessary to couple the correct amino acids to the corresponding tRNA (McClain, 1993). Holzinger et al. (2014) also observed a repressed gene expression of the tRNA-aminoacyl biosynthesis in *K. crenulatum* during desiccation. During recovery, *K. dissectum* induces the expression of both transcription- and translation-related transcripts, such as the transcription elongation factor and the ribosomal biogenesis regulatory protein (RRS1), again. Transcription elongation factors stimulate the elongation of the RNA strand by means of different mechanisms (Kim et al., 2007) while RRS1 affects the processing of pre-rRNA and ribosome assembly (Tsuno et al., 2000). Surprisingly, transcripts related to transcription and translation were up-regulated at 20°C in the desiccated filaments of *K. flaccidum*. The S1 RNA-binding domain protein (Young and Karbstein, 2011) and the U3 small nucleolar RNA-associated protein 24 (Zhang et al., 2013), for example, are involved in the ribosome assembly and WD domain containing proteins play a role in RNA processing (Stirnimann et al., 2010).

### Carbohydrate Metabolism

Many plants and algae accumulate certain sugars, such as sucrose and trehalose, in response to desiccation as these carbohydrates serve as low-molecular-weight osmolytes which counteract water potential stress (Ma et al., 2015; Fernández-Marín et al., 2016) and also have a role in ROS protection (Pommerrenig et al., 2018). Concentrating osmoprotectants in the cell establishes a negative osmotic potential, retains water within the cell, and protects both membranes and proteins (Karsten, 2012). An Antarctic strain of the chlorophyte *Trebouxia* (Sadowsky et al., 2016), for example, increased, similar to desiccation-tolerant plants (Ramanjulu and Bartels, 2002; Dinakar and Bartels, 2013), the sucrose concentration in the cell when desiccated. The same holds true for the streptophyte *Z. circumcarinatum* which enhanced starch degradation and sucrose formation upon dehydration treatment (Rippin et al., 2017). Our results for *K. dissectum* at 20°C are in agreement with these studies as the complete pathway from starch to sucrose appeared to be up-regulated when the alga is exposed to water withdrawal. Holzinger et al. (2014) reported similar findings for *K. crenulatum*. This streptophyte increased the transcript pool of sucrose synthase and sucrose phosphate synthase upon desiccation (Holzinger et al., 2014). However, no effect was visible at 5°C in *K. dissectum* which could point to a previous sucrose accumulation during the cultivation at lower temperature (e.g., frost hardening). Manabu et al. (2008) reported that cold-acclimated filaments of *K. flaccidum* contained an increased amount of sucrose, which enhanced the freezing tolerance of the alga. A similar effect is known from *Chlorella vulgaris* which accumulated raffinose in response to cold shock treatment (Salerno and Pontis, 1989).

### Membranes and Lipid Metabolism

As biomembranes are the primary target of stressors, such as low temperatures and dehydration, they are often modified in order to maintain membrane integrity and fluidity (Dinakar and Bartels, 2013; Valledor et al., 2013; Perlikowski et al., 2016). In *C. reinhardtii*, low temperature leads to two major changes in membrane composition (decrease of the lipophilic fraction and an increase in polyunsatturated fatty acids, Valledor et al., 2013). Similarly, an Antarctic ice microalga *Chlamydomonas* sp. increased the percentage of unsaturated fatty acids in the chloroplast membrane (Yi-Bin et al., 2017). A similar trend is visible in the desiccation transcriptome of *Z. circumcarinatum* as the glycero- and glycerophospholipid metabolism is activated (Rippin et al., 2017). Our data indicate that *K. dissectum* also responded with membrane modifications during water withdrawal as certain enzymes, for example the digalactosyldiacylglycerol synthase and diacylglycerol *O*-acyltransferase which are both part of the glycerolipid pathway, were up-regulated. Holzinger et al. (2014) detected an induction of the same genes in *K. crenulatum* upon dehydration. Another important protein involved in stress protection is phospholipase D which has been reported to be up-regulated in the chlorophyte *Asterochloris erici* (Gasulla et al., 2013) as well as in the streptophyte *Z. circumcarinatum* during desiccation (Rippin et al., 2017). *K. dissectum* also showed an induction of phospholipase D both at 5 and 20°C when dehydrated. Moreover, the desiccation transcriptome of *K. dissectum* suggested changes in fatty acid metabolism at 20°C. The acyl-CoA oxidase, which is part of both the β-oxidation and biosynthesis of unsaturated fatty acids, was up-regulated (Ci et al., 2015). The same holds true for two long chain acyl-CoA synthetases. These enzymes activate fatty acids and may prepare them for elongation, desaturation, lipid synthesis, and β-oxidation (Jia et al., 2016). In contrast, the acetyl-CoA carboxylase 1 is repressed upon water stress but up-regulated again during recovery. Acetyl-CoA carboxylase plays a major role in the biosynthesis of lipids as the enzyme carboxylates acetyl-CoA to form malonyl-CoA (Huerlimann and Heimann, 2013). Recovery also leads to an increase in expression of the acyl-CoA dehydrogenase, another enzyme involved in β-oxidation of fatty acids (Tan and Lee, 2016). *K. flaccidum* did not show significant regulations of these metabolic pathways.

### Cytoskeleton

In response to desiccation, tolerant plants and algae dismantle their cytoskeleton to survive the shrinkage of the cell (Proctor et al., 2007; Pressel and Duckett, 2010; Blaas and Holzinger, 2017). For example, the streptophyte alga *K. crenulatum* started to disintegrate the F-actin network after 20 min of dehydration (Blaas and Holzinger, 2017). A similar effect is probably also occurring in *K. dissectum* at both temperatures and in *K. flaccidum* at 5°C during water stress as transcripts encoding components of the cytoskeleton, such as actin and tubulin, are down-regulated. Moreover, in *K. dissectum* other proteins regulating the actin filament network are also downregulated. The expression of components of the Arp2/3 complex, which is responsible for the initiation of filament polymerization (Goley and Welch, 2006), was repressed. In addition the calcium-binding actin-bundling protein, an enzyme organizing actin filaments into dense bundles (Pikzack et al., 2005), was down-regulated. *K. flaccidum*, on the other hand, decreased the expression level of the motor protein kinesin which is an essential part of intracellular transport. The expression of the microtubule-severing enzyme katanin (Roll-Mecak and McNally, 2010) was enhanced in *K. dissectum* and *K. flaccidum* at both investigated temperatures providing a clear indication for the dismantling of the cytoskeleton during desiccation. During recovery only minor effects were visible in the transcriptomes except for *K. dissectum* at 20°C which increased the transcript pool of the above calcium-binding actin-bundling protein and two tubulin proteins.

### Protection Mechanisms

Chaperones and HSPs are an important part of the stress response as they assist with the refolding of misfolded proteins and protect them from aggregation which is generally associated with abiotic stress (Wang et al., 2004; Al-Whaibi, 2011). Heat shock, for instance, causes elevated transcript levels of HSPs in the green alga *C. reinhardtii* (Schulz-Raffelt et al., 2007; Kobayashi et al., 2014) and the red alga *Cyanidioschyzon merolae* (Kobayashi et al., 2014). HSPs are also induced by other stressors such as light stress in *Chlamydomonas* (von Gromoff et al., 1989), and desiccation stress in the green algae *A. erici* (Gasulla et al., 2013) and *Z. circumcarinatum* (Rippin et al., 2017). Our data indicate that chaperones and HSPs were also involved in the desiccation stress response of *K. dissectum* and *K. flaccidum*. HSP70, for example, was up-regulated in *K. dissectum* upon dehydration at 20°C and repressed during the second recovery phase. Dehydration had a similar effect in the mosses *Physcomitrella patens* (Tang et al., 2016) and *Sanionia uncinata* (Park et al., 2018). However, HSPs are not always accumulated during water stress, e.g., in the lichen photobiont *Trebouxia gelatinosa* (Banchi et al., 2018). The same holds true for *K. flaccidum* during desiccation treatment which increased the HSP70 transcript pool during the first recovery phase at 20°C. This trend was also observed in the moss *Bryum argenteum* 2 h after rehydration (Gao et al., 2015).

Many other chaperones also respond to desiccation stress. For example, the chaperone DnaJ was up-regulated during water stress in *K. dissectum* at 20°C and in *K. flaccidum* at 5°C. DnaJ proteins are important co-chaperones that are induced upon stress exposure (Wang et al., 2014). Similarly, *Z. circumcarinatum* increased DnaJ expression upon water withdrawal (Rippin et al., 2017).

Another important group of chaperones are ClpB proteins which are able to reverse protein misfolding caused by abiotic stressors (Lee et al., 2003). Drought stress, for example, induces the expression of ClpB proteins in *Oryza sativa* (Hu et al., 2009) and the desiccation transcriptome of *Z. circumcarinatum* showed an up-regulation of the chaperone ClpB1 (Rippin et al., 2017). Upon dehydration and rehydration at 20°C, *K. dissectum* revealed an increased transcript level of the chaperone ClpB probably to counteract the effect of protein misfolding and aggregation. At 5°C, the alga induced the expression during the first recovery phase.

Abiotic stressors, such as high light, UV radiation, cold stress, and desiccation, may cause the formation of harmful ROS making the synthesis of appropriate scavengers crucial for the survival of the cell (Cruz de Carvalho, 2008). Superoxide dismutase and catalase, for example, convert ${\text{O}}_{2}^{-}$ to H_2_O_2_ and, subsequently, to H_2_O which is no longer harmful to the cell. *K. dissectum* induced the expression of catalase at 20°C during stress treatment while *K. flaccidum* increased the transcript level at 5°C upon desiccation and decreased it immediately during the first phase of recovery. These data suggest an accumulation of ROS during stress treatment.

Plant thioredoxins are major players in the oxidative stress response (Vieira Dos Santos and Rey, 2006). We observed that thioredoxins as well as the thioredoxin reductases are up-regulated by *K. dissectum* exposed to water stress at 20°C. An elevated expression of peroxiredoxin was detected for *K. flaccidum* during the second recovery phase at 20°C. Another important cellular ROS scavenger is ascorbate which is regenerated by the dehydroascorbate reductase (Cruz de Carvalho, 2008). Upon dehydration, the transcript pool of this enzyme was increased by *K. dissectum* at 20°C as well as by *K. flaccidum* at both temperatures supporting an enrichment of ROS in the stressed filaments. Moreover, Le Martret et al. (2011) found that overexpression of the dehydroascorbate reductase and the GST in tobacco leads to an enhanced tolerance against salt and cold stress. GST is involved in the detoxification of the cell by conjugating glutathione to hydrophobic substances (Strange et al., 2001). *Z. circumcarinatum* also enhances GST expression upon desiccation stress (Rippin et al., 2017). At 20°C, the dehydration treatment resulted in an up-regulation of GST in *K. dissectum* while *K. flaccidum* induced the expression at both temperatures. ROS formation is also linked to the denaturation of biomolecules such as lipids, proteins, and nucleic acids (Cruz de Carvalho, 2008). Thus, the transcription of DNA repair proteins, such as the Nijmegen breakage syndrome 1 protein by *Z. circumcarinatum* upon desiccation, becomes necessary to minimize the destructive effect of ROS (Rippin et al., 2017). During water stress at both temperatures, *K. dissectum* induced the DNA repair protein confirming this effect. *K. dissectum* also showed an up-regulation of the phytoene desaturase, the ζ-carotene desaturase, and the carotenoid isomerase during dehydration at 20°C while the latter two are repressed at 5°C. These three enzymes are involved in the biosynthesis of carotenoids (Wurtzel et al., 2003), which protect the cell against oxidative damage (Ramel et al., 2012).

Cupins are a highly diverse protein family that may be involved in floral development, embryogenesis, as well as biotic and abiotic stress response (Dunwell et al., 2001). A cupin superfamily protein was up-regulated in *K. flaccidum* during the first recovery phase at 20°C and upon desiccation and the first rewetting at 5°C. We propose that cupins originally played a role in desiccation stress response. Possible also in plant seeds, cupins may not only play a role as storage protein, but also to achieve desiccation tolerance as in the algal ancestors of embryophytes. However, more studies are required to fully elucidate their function in *Klebsormidium*.

Early-responsive to dehydration stress genes (Kiyosue et al., 1994; Alves et al., 2011; Rai et al., 2012) have been studied and characterized in detail. These genes respond rapidly to abiotic stress, especially dehydration, and are functionally and spatially diverse (Alves et al., 2011; Rai et al., 2012). ERD2, for example, is a cytosolic HSP (Kiyosue et al., 1994) while ERD4 is a transmembrane protein, located in the chloroplast membrane, containing two tandem RNA-recognition motifs (Rai et al., 2012). During desiccation treatment at 20°C, *K. dissectum* enhanced the expression of ERD4 and another ERD protein to counteract the applied water stress. Holzinger et al. (2014) found, that in response to water withdrawal, *K. crenulatum* also induces the expression of ERD4 as well as a number of LEA proteins. LEA proteins play an essential role in the desiccation stress response as they may prevent protein aggregation and facilitate refolding (Hand et al., 2011). In response to dehydration, *K. dissectum* increased the transcription level of LEA proteins at both temperatures while *K. flaccidum* only responded at 5°C. Rippin et al. (2017) also observed an enhanced expression of LEA proteins in *Z. circumcarinatum* when subjected to desiccation. During the first recovery phase of both *Klebsormidium* species at 5°C, the LEA protein expression is further induced suggesting that these algae are still in a stressed state. The moss *Tortula ruralis* showed a similar response during desiccation and rehydration with LEA proteins being up-regulated during both treatments (Oliver et al., 2004).

## Conclusion

Overall, the applied desiccation stress has a strong effect on gene expression in both *K. dissectum* and *K. flaccidum* compared to the recovery phases. Our findings suggest that *Klebsormidium* prepares for rehydration during desiccation stress and is able to protect most of its cellular structures from damage and denaturation. Several protection mechanisms, such as osmolyte accumulation, lipid modification, ROS scavenging, and chaperones, are activated to counteract the effects of water withdrawal. However, the degree of activation differed depending on the species and temperature. Higher temperature generates a more pronounced change of expression level while cold acclimated filaments are more persistent and show a weaker reaction. We hypothesize that *Klebsormidium* obtains an increased desiccation resilience and tolerance when cultivated at low temperatures. Furthermore, *K. flaccidum* appeared to respond less to desiccation at 20°C compared to *K. dissectum*. This observation might be linked to the environmental conditions at both sampling sites. The Arctic Svalbard has a more fluctuating climate (higher summer temperature, lower winter temperature) compared to the South Shetland Islands. In addition, there exist strong environmental differences between the Arctic and Antarctica as reflected in their cold water/low temperature history (Zacher et al., 2009). Antarctica is considered to have a much longer cold water history of about 23 Mio years (Sabbe et al., 2003), compared to the “young” geological cold water history of the Arctic (ca. 2 Mio years). These striking differences in temperature history have supported the development of many endemic, cold-adapted organisms in Antarctica (e.g., Gómez et al., 2009). Our data indicate that the Antarctic *Klebsormidium* is better adapted (as measured by the lower number of genes regulated upon application of desiccation stress) in coping with desiccation than its Arctic counterpart which is likely due to a number of protection mechanisms that are permanently in place. In conclusion, we could show that environmental acclimation as well as species have a major influence on gene expression and desiccation stress response in *Klebsormidium*.

## Data Availability

All raw reads and the assemblies were submitted to the SRA database under the BioProject ID PRJNA500592.

## Author Contributions

MR and NB performed the experiments, analyzed the data, and wrote the manuscript. UK and BB conceived the study, analyzed the data, and wrote the manuscript. All authors read and approved the final manuscript.

## Conflict of Interest Statement

The authors declare that the research was conducted in the absence of any commercial or financial relationships that could be construed as a potential conflict of interest.

## Footnotes

1 http://trinotate.github.io/

2 http://transdecoder.github.io/

## Supplementary Material

FIGURE S1 KEGG map overview showing all the mapped annotated KO IDs for *K. dissectum*.

FIGURE S2 KEGG map overview showing all the mapped annotated KO IDs for *K. flaccidum*.

TABLE S1 Statistics of the sequencing outcome. The number of read pairs, quality pairs, quality merged reads, quality single reads, and the mapping efficiency on the assembled transcripts are given for each replicate. Kd and Kf indicate *K. dissectum* and *K. flaccidum*. Five and 20 indicate the temperatures at which the experiment was performed. C, control cells; D, desiccated cells; HI, rewetted sample I (1 h); HII, rewetted sample II (24 h after the start of experiment).

TABLE S2 GO enrichment analysis. All GO terms enriched are shown for all comparisons.

TABLE S3 Selected genes regulated in *K. dissectum* and *K. flaccidum* upon desiccation and/or rehydration.
