# Supplementary figures and images for: Cold Acclimation Improves the Desiccation Stress Resilience of Polar Strains of Klebsormidium (Streptophyta)
Source: Front Microbiol. 2019 Aug 6;10:1730. doi: 10.3389/fmicb.2019.01730 (PMC6691101; doi:10.3389/fmicb.2019.01730)

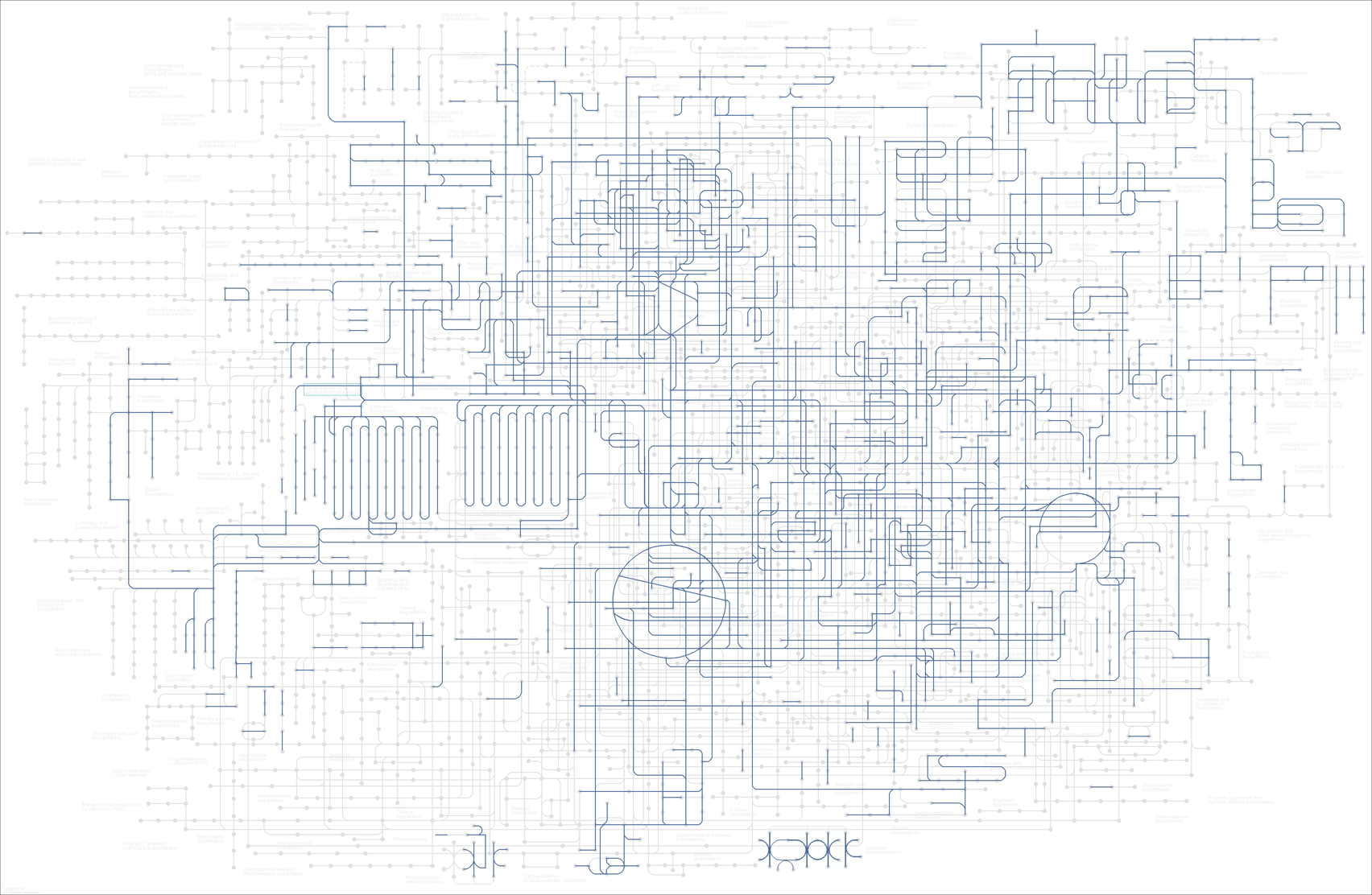

Supplement: FIGURE S1 — KEGG map overview showing all the mapped annotated KO IDs for K. dissectum. [file Image_1.png]

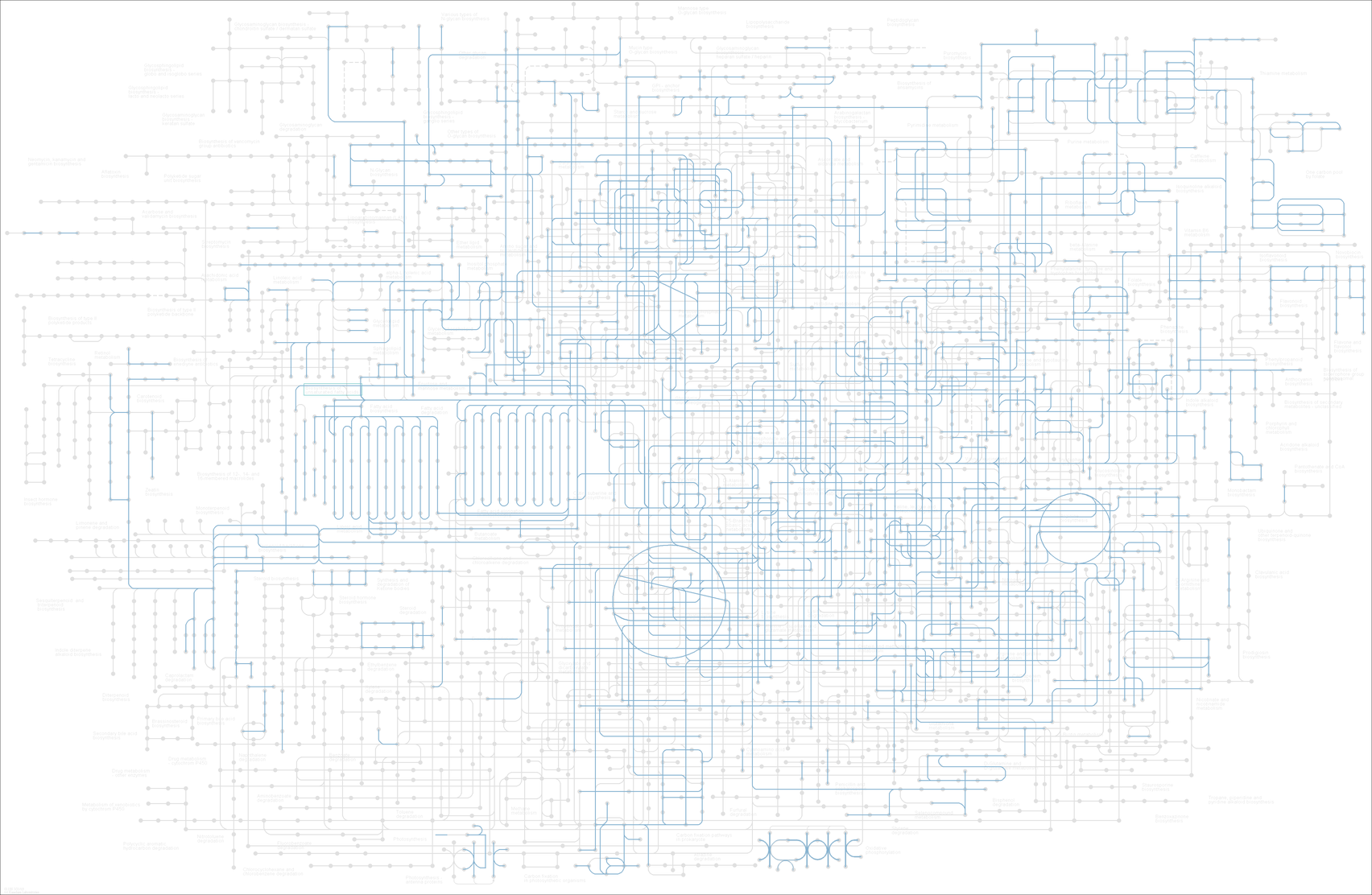

Supplement: FIGURE S2 — KEGG map overview showing all the mapped annotated KO IDs for K. flaccidum. [file Image_2.png]
