# Supplementary material for: Cold Acclimation Improves the Desiccation Stress Resilience of Polar Strains of Klebsormidium (Streptophyta)
Source: Front Microbiol. 2019 Aug 6;10:1730. doi: 10.3389/fmicb.2019.01730 (PMC6691101; doi:10.3389/fmicb.2019.01730)
Supplement: TABLE S3 — Selected genes regulated in K. dissectum and K. flaccidum upon desiccation and/or rehydration. [file Table_3.docx]

**Table 3:** Selected genes regulated upon desiccation and/or rehydration The ID of selected regulated transcripts from K. dissectum od K. flaccidum as well as the ID of the most similar K. nitens gene are given. The log2 fold range is given for pairwise comparisons: CD: control – desiccated samples; DRI: desiccated – recovery I samples; RIRII: recovery I – recovery II samples at 5° and 20°C.

| **Photosynthesis** |  |  |  |  |  |  |  |  |  |
| --- | --- | --- | --- | --- | --- | --- | --- | --- | --- |
|  | **K. nitens ID** | **Annotation** | **E value** | **20_CD** | **20_DRI** | **20_RIRII** | **5_CD** | **5_DRI** | **5_RIRII** |
| **K. dissectum ID** |  |  |  |  |  |  |  |  |  |
| TR8488\|c0_g2_i1 | kfl00036_0310 | Photosystem I subunit (psaN) | 2,06E-21 | -11,3 | - | - | - | - | - |
| TR41475\|c0_g1_i1 | kfl00614_0030 | Photosystem II 33 kDa polypeptide (psbO) | 0 | -6,2 | - | 4,0 | - | - | - |
| TR33793\|c0_g1_i1 | kfl00239_0120 | Photosystem II oxygen-evolving enhancer protein II-1 (psbP1-1) | 2,04E-115 | -9,3 | - | - | - | - | - |
| TR40824\|c0_g1_i1 | kfl00191_0030 | Photosystem II oxygen-evolving enhancer protein II-2 (psbP1-2) | 3,43E-151 | -3,3 | - | 4,6 | -2,4 | - | - |
| TR47358\|c0_g1_i2 | kfl00053_0120 | Photosystem II subunit (psb27) | 0 | -2,1 | - | 3,8 | - | - | - |
| TR7748\|c0_g2_i1 | kfl00517_0040 | Light-harvesting complex I chlorophyll a/b binding protein 2 (LHCA2) | 0 | -8,3 | - | - | - | - | - |
| TR34316\|c0_g1_i2 | kfl00422_0020 | Light harvesting chlorophyll a b-binding protein (LHCB3) | 0 | -5,2 | - | 3,9 | - | - | - |
| TR7433\|c0_g1_i2 | kfl00214_0040 | Magnesium chelatase subunit (CHLD) | 0 | -4,3 | - | 4,3 | - | - | - |
| TR453\|c0_g2_i2 | kfl00161_0080 | Early light-induced protein (ELIP1a.2) | 3,01E-123 | 4,2 | - | 5,5 | -2,8 | - | - |
| TR34827\|c0_g1_i2 | kfl00052_0260 | Early light-induced protein (ELIP2.2) | 1,30E-134 | 3,7 | 2,9 | - | -6,8 | - | - |
| **K. flaccidum ID** |  |  |  |  |  |  |  |  |  |
| TR5381\|c0_g1_i1 | kfl00161_0070 | Early light-induced protein (ELIP1a.1) | 1,21E-126 | - | - | 8,1 | 4,5 | - | - |
| TR1303\|c0_g1_i3 | kfl00161_0080 | Early light-induced protein (ELIP1a.2) | 4,97E-63 | 5,0 | - | - | 5,1 | - | - |
| TR21415\|c0_g3_i1 | kfl00110_0040 | Early light-induced protein (ELIP1b) | 1,73E-102 | - | - | 5,4 | 5,8 | - | - |
| TR39583\|c0_g2_i1 | kfl00211_0160 | Early light-induced protein (ELIP1d) | 3,59E-116 | - | - | 8,4 | 5,2 | - | - |
| TR38255\|c0_g1_i1 | kfl00592_0040 | Early light-induced protein (ELIP2.3) | 2,7E-101 | - | - | 8,7 | - | - | - |
| **Cell Cycle** |  |  |  |  |  |  |  |  |  |
| **K. dissectum ID** |  |  |  |  |  |  |  |  |  |
| TR36659\|c0_g1_i1 | kfl00039_0170 | Chromosome condensation complex Condensin, subunit H | 0 | -12,0 | - | - | - | - | - |
| TR5178\|c0_g1_i1 | kfl00026_0260 | Spindle assembly checkpoint protein | 2,50E-122 | -11,0 | - | - | - | - | - |
| TR11732\|c0_g1_i1 | kfl00220_0050 | Leishmanolysin-like peptidase | 0 | -5,2 | - | - | -4,1 | - | - |
| TR19558\|c0_g1_i7 | kfl00080_0110 | Helicase conserved C-terminal domain containing protein | 0 | -9,0 | - | 10,2 | - | - | - |
| TR50075\|c0_g1_i1 | kfl00100_0250 | DNA primase, large subunit | 0 | -5,1 | - | - | - | - | - |
| TR9153\|c0_g2_i1 | kfl00061_0280 | DNA polymerase epsilon subunit 1 (POLE1) | 0 | -4,8 | - | - | - | - | - |
| TR27196\|c2_g1_i1 | kfl00422_0010 | DNA replication licensing factor, MCM2 component | 0 | -7,5 | - | - | - | - | - |
| TR38994\|c0_g2_i1 | kfl00029_0270 | DNA replication licensing factor, MCM6 component | 0 | -7,0 | - | - | - | - | - |
| TR13055\|c0_g1_i1 | kfl00481_0090 | DNA replication licensing factor, MCM7 component | 0 | -6,7 | - | - | - | - | - |
| **K. flaccidum ID** |  |  |  |  |  |  |  |  |  |
| TR14942\|c0_g2_i1 | kfl00814_0010 | Centromere-associated protein HEC1 | 0 | -3,7 | - | - | -2,5 | - | - |
| TR13748\|c0_g2_i1 | kfl00106_0220 | TPR domain containing protein | 0 | - | - | - | -8,9 | - | - |
| TR46436\|c0_g2_i2 | kfl00046_0010 | Pre-initiation complex, subunit CDC6, AAA+ superfamily ATPase | 0 | - | - | - | - | -3,0 | - |
| TR12145\|c0_g1_i5 | kfl00203_0160 | D- helicase | 0 | -8,8 | - | - | - | - | - |
| TR75\|c0_g1_i1 | kfl00422_0010 | D- replication licensing factor, MCM2 component | 0 | -4,6 | - | - | - | -3,0 | - |
| TR34347\|c0_g2_i1 | kfl00788_0010 | D- replication licensing factor, MCM3 component | 0 | -2,6 | - | - | - | -2,4 | - |
| TR44161\|c0_g1_i1 | kfl00184_0010 | D- replication licensing factor, MCM5 component | 0 | -9,4 | - | - | - | - | - |
| TR48102\|c0_g1_i1 | kfl00029_0270 | D- replication licensing factor, MCM6 component | 0 | -3,7 | - | - | - | -2,3 | - |
| **Transkription/ Translation** |  |  |  |  |  |  |  |  |  |
| **K. dissectum ID** |  |  |  |  |  |  |  |  |  |
| TR32329\|c0_g1_i1 | kfl00592_0020 | U3 small nucleolar RNA-associated protein 10 (UTP10, HEATR1) | 0 | - | 3,2 | - | - | - | - |
| TR13905\|c0_g2_i3 | kfl00507_0030 | Transcription elongation factor | 0 | - | 9,7 | - | - | - | - |
| TR21130\|c0_g1_i2 | kfl00088_0080 | Splicing factor, arginine/serine-rich | 0 | -11,3 | 10,1 | - | - | - | - |
| TR12893\|c1_g1_i9 | kfl00264_0020 | RNA recognition motif (RRM)-containing protein | 0 | - | 8,8 | - | - | - | - |
| TR40135\|c0_g1_i7 | kfl00085_0420 | Ribosomal biogenesis regulatory protein (RRS1) | 0 | - | 2,3 | - | - | - | - |
| TR29665\|c0_g1_i1 | kfl00215_0120 | Aspartyl/Asparaginyl-tRNA synthetase, class IIb | 0 | -3,4 | - | 5,1 | - | - | - |
| TR28640\|c0_g1_i1 | kfl00444_0100 | Methionyl-tRNA synthetase | 0 | -6,0 | - | 10,9 | - | - | - |
| TR1930\|c0_g1_i6 | kfl00079_0090 | Phenylalanyl-tRNA synthetase, class IIc, beta subunit | 0 | -7,6 | - | - | - | - | - |
| TR13948\|c0_g1_i1 | kfl00106_0070 | Valyl-tRNA synthetase | 0 | -5,4 | - | 5,3 | - | - | - |
| TR44078\|c0_g2_i1 | kfl00017_0480 | N-acetyltransferase 10 | 0 | - | 2,1 | - | - | - | - |
| **K. flaccidum ID** |  |  |  |  |  |  |  |  |  |
| TR49350\|c0_g1_i14 | kfl00054_0210 | S1 R- binding domain containing protein | 1,15E-174 | 8,4 | - | - | - | - | - |
| TR13930\|c0_g1_i1 | kfl00346_0050 | U3 small nucleolar RNA-associated protein 24 (UTP24, FCF1) | 4,42E-130 | 9,5 | - | - | - | - | - |
| TR8236\|c0_g1_i2 | kfl00065_0230 | WD domain containing protein | 0 | 8,6 | - | - | - | - | - |
| TR27840\|c0_g1_i3 | kfl00444_0090 | N-terminal acetyltransferase | 0 | 8,9 | - | - | 2,6 | - | - |
| **Carbohydrate Metabolism** |  |  |  |  |  |  |  |  |  |
| **K. dissectum ID** |  |  |  |  |  |  |  |  |  |
| TR19261\|c1_g1_i3 | kfl00228_0110 | alpha-Amylase | 0 | 6,9 | - | - | - | - | - |
| TR14575\|c1_g1_i2 | kfl00081_0270 | beta-Amylase | 0 | 9,0 | - | - | - | - | - |
| TR28615\|c0_g4_i2 | kfl00063_0030 | 4-alpha-Glucanotransferase | 0 | 2,4 | - | - | - | - | - |
| TR39144\|c1_g1_i2 | kfl00085_0350 | Hexokinase | 0 | 2,3 | - | - | - | - | - |
| TR26404\|c0_g3_i7 | kfl00016_0540 | Phosphoglucomutase | 0 | 2,0 | - | - | - | - | - |
| TR32865\|c0_g1_i2 | kfl00026_0070 | Sucrose phosphorylase | 0 | 4,9 | - | - | - | - | - |
| TR401\|c1_g1_i9 | kfl00065_0210 | Sucrose synthase | 0 | 7,8 | - | - | - | - | - |
| TR15248\|c0_g1_i1 | kfl00739_0060 | Sucrose-phosphate synthase | 0 | 9,2 | - | - | - | - | - |
| TR28617\|c0_g2_i3 | kfl00228_0150 | Sucrose-phosphatase | 0 | 4,8 | - | - | - | - | - |
| **Membranes/Lipid Metabolism** |  |  |  |  |  |  |  |  |  |
| **K. dissectum ID** |  |  |  |  |  |  |  |  |  |
| TR19566\|c1_g1_i5 | kfl00011_0330 | Phospholipase D | 0 | 6,9 | - | - | 10,2 | - | - |
| TR19302\|c2_g1_i21 | kfl00342_0160 | Phosphoethanolamine N-Methyltransferase (PEAMT) | 0 | 4,4 | - | - | 2,5 | - | - |
| TR47741\|c0_g2_i2 | kfl00030_0330 | digalactosyldiacylglycerol (DGDG) synthase | 0 | 3,4 | - | - | - | - | - |
| TR47379\|c0_g1_i2 | kfl00110_0050 | diacylglycerol O-acyltransferase | 0 | 5,0 | - | - | - | - | - |
| TR20319\|c0_g1_i8 | kfl00025_0100 | Membrane bound O-acyl transferase family protein (MBOAT) | 0 | 9,6 | - | - | - | - | - |
| TR15216\|c0_g1_i4 | kfl00041_0220 | Acyl-CoA oxidase | 0 | 3,2 | - | - | - | 2,8 | - |
| TR9129\|c0_g1_i1 | kfl00230_0070 | Acyl-CoA dehydrogenase | 0 | - | - | 9,1 | - | - | - |
| TR9045\|c0_g1_i1 | kfl00034_0420 | Acetyl-CoA carboxylase 1 | 0 | -11,6 | - | 9,2 | - | - | - |
| TR51057\|c0_g1_i20 | kfl00274_0140 | Long-Chain Acyl-CoA Synthetase | 0 | 6,5 | - | - | - | - | - |
| TR41415\|c1_g1_i2 | kfl00027_0510 | Long-Chain Acyl-CoA Synthetase, plastid | 0 | 5,1 | - | - | - | - | - |
| **Cytoskeleton** |  |  |  |  |  |  |  |  |  |
| **K. dissectum ID** |  |  |  |  |  |  |  |  |  |
| TR4773\|c0_g1_i1 | kfl00105_0040 | Actin-related protein Arp2/3 complex, subunit ARPC3 | 1,58E-89 | -4,0 | - | - | - | - | - |
| TR17297\|c0_g1_i2 | kfl00231_0040 | Calcium-binding actin-bundling protein (fimbrin/plastin), EF-Hand protein superfamily | 0 | -5,8 | - | 5,7 | - | - | - |
| TR23564\|c0_g2_i1 | kfl00032_0390 | Tubulin | 0 | -3,1 | - | 5,3 | - | - | - |
| TR15253\|c0_g2_i2 | kfl00501_0070 | gamma-Tubulin | 0 | -4,3 | - | 5,0 | - | - | - |
| TR28702\|c0_g1_i1 | kfl00044_0370 | delta-Tubulin | 0 | -2,3 | - | - | - | - | - |
| TR34366\|c0_g1_i2 | kfl00223_0140 | epsilon-Tubulin | 6,28E-47 | -10,3 | - | - | -2,8 | - | - |
| TR3865\|c0_g1_i1 | kfl00815_0030 | beta-Tubulin folding cofactor C | 0 | - | - | - | -7,5 | - | - |
| TR40792\|c0_g1_i3 | kfl00366_0060 | Katanin p60 ATPase-containing subunit, putative | 0 | 2,2 | - | - | - | - | - |
| **K. flaccidum ID** |  |  |  |  |  |  |  |  |  |
| TR49533\|c0_g1_i1 | kfl00570_0030 | Actin and related proteins | 0 | - | - | - | -2,9 | - | - |
| TR5027\|c0_g2_i1 | kfl00519_0020 | Kinesin-like protein | 0 | - | - | - | -9,3 | - | - |
| TR16574\|c0_g1_i4 | kfl00060_0070 | Dynamin family protein | 0 | - | - | - | -4,8 | - | - |
| TR50192\|c1_g1_i12 | kfl00366_0060 | Katanin p60 ATPase-containing subunit, putative | 0 | - | - | - | 2,3 | - | - |
| **Drought Stress Response** |  |  |  |  |  |  |  |  |  |
| **K. dissectum ID** |  |  |  |  |  |  |  |  |  |
| TR10799\|c0_g2_i1 | kfl00440_0020 | Heat shock protein 70 (Hsp 70) family protein | 0 | 4,0 | - | -2,8 | - | - | - |
| TR21756\|c0_g1_i4 | kfl00042_0120 | Chaperone DnaJ-domain superfamily protein | 1,15E-165 | 3,5 | - | - | - | - | - |
| TR19284\|c0_g1_i8 | kfl00027_0040 | ATP-dependent chaperone ClpB | 0 | 5,4 | 3,2 | -2,5 | - | 3,0 | - |
| TR13733\|c0_g1_i2 | kfl00027_0240 | Catalase | 0 | 10,2 | - | - | - | - | - |
| TR27815\|c0_g1_i7 | kfl00386_0040 | Putative NADPH-dependent thioredoxin reductase | 0 | 2,3 | - | - | - | - | - |
| TR28643\|c0_g1_i5 | kfl00121_0210 | Thioredoxin superfamily protein | 1,58E-97 | 11,2 | - | - | - | - | - |
| TR14779\|c0_g1_i7 | kfl00025_0120 | Dehydroascorbate reductase | 2,00E-170 | 10,0 | - | - | - | - | - |
| TR27837\|c0_g1_i2 | kfl00270_0040 | Glutathione S-transferase | 1,66E-122 | 8,3 | - | - | - | - | - |
| TR45259\|c0_g1_i7 | kfl00040_0200 | Glutathione transferase | 0 | 4,8 | - | - | - | - | - |
| TR33920\|c0_g1_i3 | kfl00101_0120 | DNA repair protein | 0 | 4,0 | - | - | 2,2 | - | - |
| TR12924\|c2_g3_i9 | kfl00012_0080 | zeta-Carotene desaturase | 0 | 15,0 | - | - | -15,4 | 15,3 | - |
| TR19264\|c0_g1_i14 | kfl00085_0130 | Phytoene dehydrogenase, phytoene desaturase | 5,28E-177 | 10,5 | - | - | - | - | - |
| TR6489\|c0_g1_i1 | kfl00043_0200 | FAD/NAD(P)-binding oxidoreductase family protein, putative carotenoid isomerase | 0 | 6,0 | - | 2,5 | -2,9 | - | - |
| TR20556\|c0_g2_i3 | kfl00029_0020 | Early-responsive to dehydration stress (ERD) family protein | 0 | 8,5 | - | - | - | - | - |
| TR28621\|c0_g1_i4 | kfl00551_0030 | Early-responsive to dehydration stress protein (ERD4) | 0 | 4,9 | - | - | - | - | - |
| TR40127\|c0_g2_i2 | kfl00378_0060 | Late embryogenesis abundant protein | 5,52E-128 | 7,8 | - | -3,3 | 2,6 | 2,8 | - |
| **K. flaccidum ID** |  |  |  |  |  |  |  |  |  |
| TR55653\|c0_g2_i9 | kfl00430_0080 | Heat shock protein 70 (Hsp 70) family protein | 0 | - | 11,9 | - | - | - | -10,8 |
| TR15839\|c0_g1_i4 | kfl00042_0120 | Chaperone DnaJ-domain superfamily protein | 7,6E-66 | - | - | - | 7,1 | - | - |
| TR7911\|c0_g1_i1 | kfl00241_0050 | ATP-dependent chaperone | 9,6E-24 | 8,6 | - | - | - | - | - |
| TR21212\|c0_g2_i1 | kfl00287_0090 | Catalase | 0 | - | - | - | 10,9 | -10,8 | - |
| TR7856\|c0_g1_i3 | kfl00014_0250 | Peroxiredoxin | 2,86E-116 | - | - | 8,8 | - | - | - |
| TR27896\|c2_g3_i3 | kfl00025_0120 | Dehydroascorbate reductase | 3,58E-162 | 6,4 | - | - | 3,9 | - | - |
| TR22675\|c0_g1_i1 | kfl00166_0100 | Glutathione S-transferase family protein | 4,02E-171 | 2,7 | - | - | 2,0 | - | - |
| TR56584\|c0_g4_i4 | kfl00003_0220 | Cupin superfamily protein | 9,35E-73 | - | 11,8 | -11,9 | 5,4 | 2,0 | - |
| TR3299\|c0_g3_i1 | kfl00062_0210 | Late embryogenesis abundant protein | 1,15E-161 | - | - | - | 2,6 | 2,3 | - |
